# Renal injury following long-term exposure to carbon disulfide: analysis of a case series

**DOI:** 10.1186/s12882-019-1553-1

**Published:** 2019-10-17

**Authors:** Yongqin Yan, Cheng Wang, Zhiyong Zheng, Lijuan Qu, Dehua Zeng, Min Li

**Affiliations:** 1 Department of Pathology, 900 Hospital of the jiont logistic, Fuzhou, China; 2 Yibin changning county hospital of traditional Chinese medicine, Yibin, Sichuan Province China

**Keywords:** Glomeruli, Mesangial, Idiopathic, Nodular mesangial hyperplasia/sclerosis, Carbon disulfide

## Abstract

**Background:**

To investigate the clinicopathological characteristics of renal damage caused by long-term exposure to carbon disulfide (CS2) in nine patients.

**Methods:**

All the patients underwent ultrasound-guided renal biopsy. All specimens were examined by light microscopy and immunohistochemistry (IHC). Samples form one patient were further analyzed using transmission electron microscopy.

**Results:**

Similar pathological changes were observed in all patients, but the degrees of lesions were different. All cases had moderate to severe nodular mesangial hyperplasia; among these, type “Kimme1stie1-Wi1son” (K-W nodule for short) was observed in four cases, type “K - W nodule” refer to nodular hyperplasia of mesangial membrane like letter K or W. four cases had proliferative extracapillary glomerulonephritis (GN), while there were no concomitant changes in one patient. Besides, six cases had diffuse basement membrane thickening, focal segmental sclerosis or bulbar sclerosis; two cases had diffuse glomerular sclerosis, and one case had focal segmental capillary hyperplasia. Moreover, all patients had renal tubular atrophy/interstitial fibrosis with less to moderate chronic inflammatory cell infiltration, as well as renal arteriosclerosis. IHC showed that the depositions of IgA, IgM, C3d, C4d, C1q and Fib were not specific; while IgG, type III collagen, Fibronectin, Amyloid A, Igκ, Igλ, HBsAg and HBcAg were all negative.

**Conclusion:**

Diffuse nodular mesangial hyperplasia/sclerosing glomerular nephropathy is characterized by nodular mesangial hyperplasia with type “K-W nodules” formation, which we speculate is a special pathological manifestation of renal damage caused by carbon disulfide (CS2).

## Background

Carbon disulfide (CS2) is a colorless volatile chemical solvent, widely used in industry as viscose fiber, glass paper, vulcanized rubber, carbon tetrachloride, and pesticide. CS2 is highly toxic and can lead to acute or chronic poisoning [1]. Inhalation is the major route of exposure to CD followed by skin/eye contact and ingestion. Acute intoxication can further lead to neuropsychiatric symptoms, severe brain edema, and in some severe cases coma and death. Chronic poisoning leads to neurological damage (e.g. mental symptoms, polyneuritis, neuropathy, etc.) and cardiovascular system injury (e.g. brain, retina, renal and coronary arteriosclerosis, blood cholesterol increase, etc.) [2] . Moreover, it has been reported that chronic poisoning can cause renal function damage; pathological changes mainly include glomerular nodular mesangial hyperplasia [3].

Here we presented a series of nine cases with CS2 toxic nephropathy. The clinical and pathological characteristics of these cases were summarized, and related literature was reviewed.

## Materials and methods

### Clinical data

Renal specimens were collected from nine patients with heavy proteinuria who underwent renal puncture biopsy between January 2013 and December 2014. Samples were analyzed at the Department of Pathology, Dong fang Hospital. The clinical history data included gender, age, course of disease (the number of days from proteinuria to renal puncture), and blood pressure (systolic pressure of 140 = mmHg and/or diastolic pressure of 90 mmHg were defined as hypertension). Laboratory indicators before renal penetration included: quantitative/24 h urine protein, microscopic haematuria, creatinine, serum creatinine (SCR, normal 53 ~ 124umol/L), urea nitrogen (BUN, normal tendency of 2.9 ~ 8.9 L), Fasting blood - glucose (FBG, normal 3.89 ~ 6.11 mmol/L), autoantibody (anti cardiolipin, ANA, anti dsDNA, anti SSA, SSB resistance, resistance to SM, ENA resistance, anti GBM antibody, c - ANCA and p - ANCA), immunoglobulin and complement (IgG, IgM and IgA, C3 and C4), five hepatitis B virus (HBsAg, HBeAg, anti - HBs, anti - HBe, anti - HBc).

### IHC and staining

All specimens of renal puncture were fixed in 4% neutral buffered formalin, embedded in paraffin and then cut in 5 μm sections. Samples were analyzed using light microscopy (one case was analyzed using transmission electron microscopy) and IHC.

Samples were stained with one of the following methods: HE staining, PAS staining, PAM – Masson staining and Congored staining. For IHC, samples were incubated with the following antibodies: IgG, IgA, IgM, C3d, C4d, C1q, fibrinogen (Fib), collagen type III, fibronectin, amyloid A and Ig kappa, Ig lambda, HBsAg and HBcAg dyeing predominate; C3d and C4d were acquired form Abcam company and Biomedica company, respectively; HBsAg, HBcAg and EliVision kit were bought from Fuzhou company; while all others antibodies were purchased from Dako company.

For antigen repair, the following antibodies were used IgG, IgA, IgM, C3d, C4d, C1q, Fib, Amyloid A, Ig common wealth, and Ig mind chains. Briefly, samples were treated with antibodies, mixed with 0.01 mol/L pH 6.0 citrate buffer at high-temperature and high-pressure repair of the antigen, plus 0.4% gastric enzyme (purchased from Anresco) for digestion for 5 min [4]_**.**_ After HBsAg and HBcAg staining was repaired with high-temperature high-pressure antigen, 0.05% 24-type protease was added for 7 min [5]_**.**_ Collagen type II (HWD1.1) and fibronectin in only 0.05% of 24 type protease digestion for 10 min.

### Pathological observation methods

The pathological diagnostic criteria were classified according to the WHO glomerular disease classification published in 1995 [6] and diabetic nephropathy classification principle of the international association of nephropathy from 2010 [7]. The tubulointerstistitial lesion (TIL) scores [8] were classified as: score 0, < 6% lesion; score 1, 6~25% lesion; score 2, 26%~ 50% lesion; score 3, > 50% lesion. The numbers of arteriosclerosis (SAS) were analyzed as follows: 0 points for non-sclerosis, 1 for 1–25% arteriosclerosis; two for 26% ~ 50% arteriosclerosis; three for > 50% arteriosclerosis; 1 point was added if the intima of the interlobular artery thickens beyond the mesenchymal. IHC semi-quantitative scoring [9] was analyzed according to the following criteria: positive range accounted for < 5% of total glomerular area; 1 for 6%~ 25% area; 2 for 26%~ 50% area; 3 for > 50% area. The color intensity was 1, 2 and 3. A positive score (0~9 points) was obtained when the score of positive range and color intensity was multiplied.

## Results

### Clinical data

Clinical data of patients are shown in Table 1. Patients were all male, aged between 30 and 37 (median age 33; working age 11 to 17 years (mean 13.2 years)); course 1~150 days, median course 16 days. Large amounts of proteinuria and microscopic hematuria were found in nine cases; five cases had increased SCR and BUN, and 2 cases had hypertension. In addition, chronic renal failure (CRF) was observed in 4 patients. Psychiatric symptoms (anxiety, paranoia and personality changes) and neurological symptoms (lower limb pain, claudication and slow nerve conduction in extremities) were found in 3 cases. The fasting blood – glucose (FBG) of 9 patients were normal, which indicated that those patients were not diabetic. Complete set of autoantibodies, immunoglobulin, complement and serum HBsAg, HBeAg and anti-HBc were negative. Responses to treatment showed hormonal resistance in nine cases. Proteinuria persisted for 14 to 50 months of follow-up, and two cases developed ESRD.

**Table 1:** Clinical data

Serial number	Age	working age (year)	Course (day)	Clinical diagnosis	Proteinuria (mg /24 h)	Hematuria (cells/HP)	SCr (umol/L)	BUN (mmol/L)	Blood pressure (mmHg)	Glucocorticoid efficacy	Follow-up (month)	Fasting blood - glucose	ESRD	Nerve damage
1	31	14	1	Chronic renal failure	2344.6	14	153	10	100/80	SR	14	6.08	no	no
2	36	14	7	Chronic renal failure	392	11	126	6.4	120/80	SR	15	4.5	no	no
3	34	14	3	Chronic renal failure	8228.9	24	248	10.8	140/80	SR	14	4.7	yes	no
4	37	17	7	Proteinuria to be examined	778.4	4	128	9.1	150/90	SR	10	5.13	no	no
5	30	14	90+	Chronic renal failure	3932.8	4	231	12.7	135/90	SR	16	6.0	no	no
6	35	11	150+	Proteinuria to be examined	2917.2	5	118	10.7	160/100	SR	50	4.3	yes	yes
7	30	12	60+	Proteinuria to be examined	1900	7	66	4.2	130/80	SR	22	5.5	no	yes
8	33	11	23	Proteinuria to be examined	1410	17	90	5.1	135/80	SR	23	5.0	no	no
9	34	12	150+	Proteinuria to be examined	2185.6	6	65	4.6	140/88	SR	47	5	no	yes

### Light microscopy

**Fig. 1:**
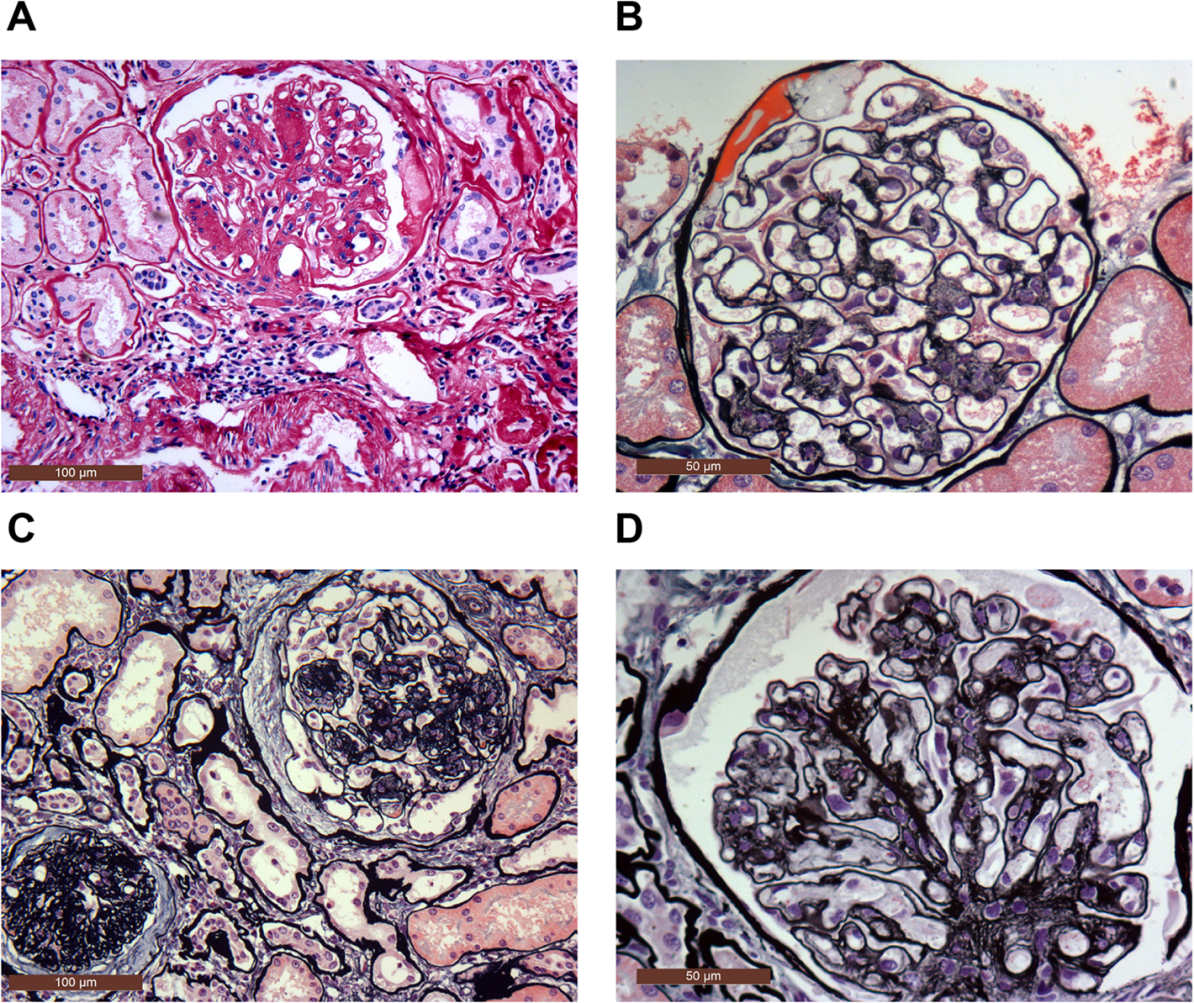
**a** Glomerular nodular mesangial proliferation, K-W nodular formation, small arteriosclerosis (PSA, × 200). **b** Glomerular nodular mesangial cells and mesangial stromal hyperplasia, vascular haptics basement membrane uniform thickening, stiffness, cladman’s capsule wall attached to the half-moon protein material (PSAM-Masson,× 400). **c** Glomerular nodular mesangial cells and mesangial stromal hyperplasia / sclerosing, fibrous crescent formation, focal sclerosis (PSAM-Masson, × 200). **d** Glomerular nodular mesangial cells and mesangial stromal hyperplasia, vascular haptics basement membrane double-orbital (PSAM-Masson, × 400)

Similar pathological changes were observed in nine patients; nevertheless, the degrees of lesions were different **(**Table 2, Fig. 1). All cases showed moderate to severe nodular mesangial hyperplasia; type “K - W nodule” was observed in four cases, fSour cases had proliferative extracapillary glomerulonephritis (GN) (2 cases had fibrous crescent GN and 2 cases cellular crescent GN), while there were no concomitant changes in one patient. Besides, six cases had diffuse basement membrane thickening, focal segmental sclerosis or bulbar sclerosis; two cases had diffuse glomerular sclerosis, and one case had focal segmental capillary hyperplasia. Moreover, all patients had renal tubular atrophy/interstitial fibrosis with less to moderate chronic inflammatory cell infiltration, as well as renal arteriosclerosis (Table 2).

**Table 2:** Pathological features

Serial number	Type of glomerular lesions	Lesion score		IHC positive score						
		TIL	SAS	IgG	IgM	IgA	C3d	C4d	C1q	Fib
1	Diffuse nodular mesangial sclerosis	3	2	0	3	1	2	3	2	1
2	Diffuse nodular mesangial proliferation, focal type I membrane proliferation, focal segment / pelvic glomerulosclerosis	1	2	0	2	2	4	2	1	2
3	Diffuse nodular sclerosis with type I membrane proliferation, focal crescent, focal segmental sclerosis	3	2	0	2	0	4	2	2	1
4	Diffuse nodular mesangial proliferation, focal type I membrane proliferation, focal segment / pelvic glomerulosclerosis	2	3	0	2	0	1	1	1	1
5	Diffusesclerosingglomerulonephritis	3	3	0	4	0	1	1	1	1
6	Diffuse nodular mesangial proliferation with type I membrane proliferation, focal segment / sclerosis	2	3	0	2	0	2	2	1	1
7	Diffuse nodular mesangial proliferation	1	3	0	1	2	4	1	1	1
8	Diffuse nodular mesangial sclerosis	1	2	0	1	1	2	1	1	1
9	Diffuse nodular mesangial proliferation with segment I type membrane proliferation	2	2	0	1	0	4	4	4	1

### IHC and electron microscopy

**Fig. 2:**
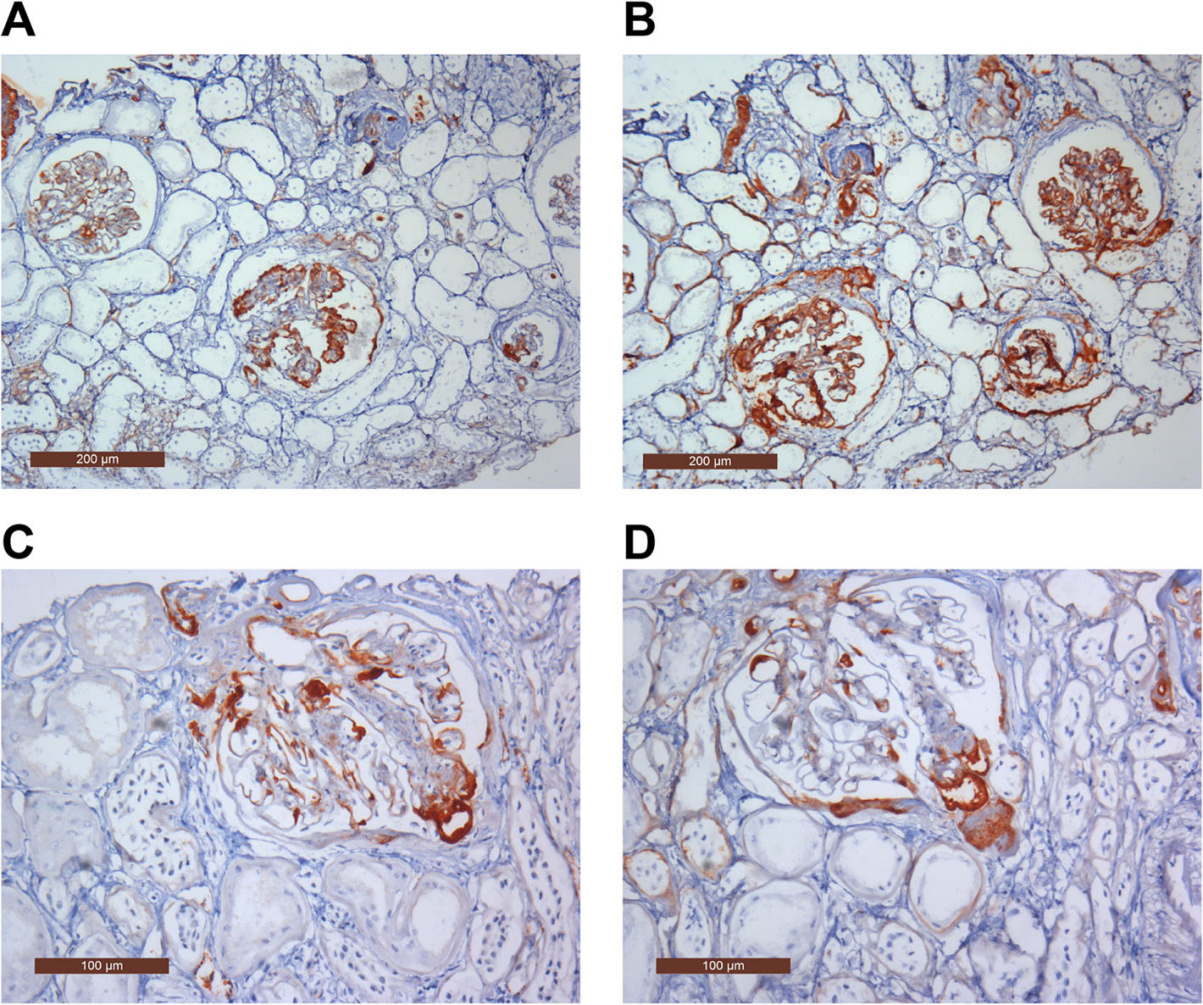
**a** IgM was highly expressed in the segmental sclerosis and weakly expressed in the mesangial region (EliVision, × 100). **b** C3d was highly expressed in the mesangial region and weakly expressed in the vascular haptics (EliVision, × 100). **c** C4d deposition in the segment hardening (EliVision, × 200). **d** Clq deposition in the hardened section (EliVision, × 200)

In all patients, IgM were strongly expressed in segmental sclerosis and weakly expressed in the mesangial region; among these, 4 cases had a small amount of mesangial IgA deposition in focal segmental region. C3d was strongly expressed in diffuse mesangial region and weakly expressed in the vascular loop. C4d, C1q and Fib were all expressed in segmental sclerotic region; nevertheless, IgM and IgA, IgG, C4d, C1q and Fib were no specificity of sedimentary. Type III collagen and Fibronectin, Amyloid A and Ig kappa, Ig lambda predominate, both for HBsAg and HBcAg were negative (Table 2**,** Fig. 2).

**Fig. 3:**
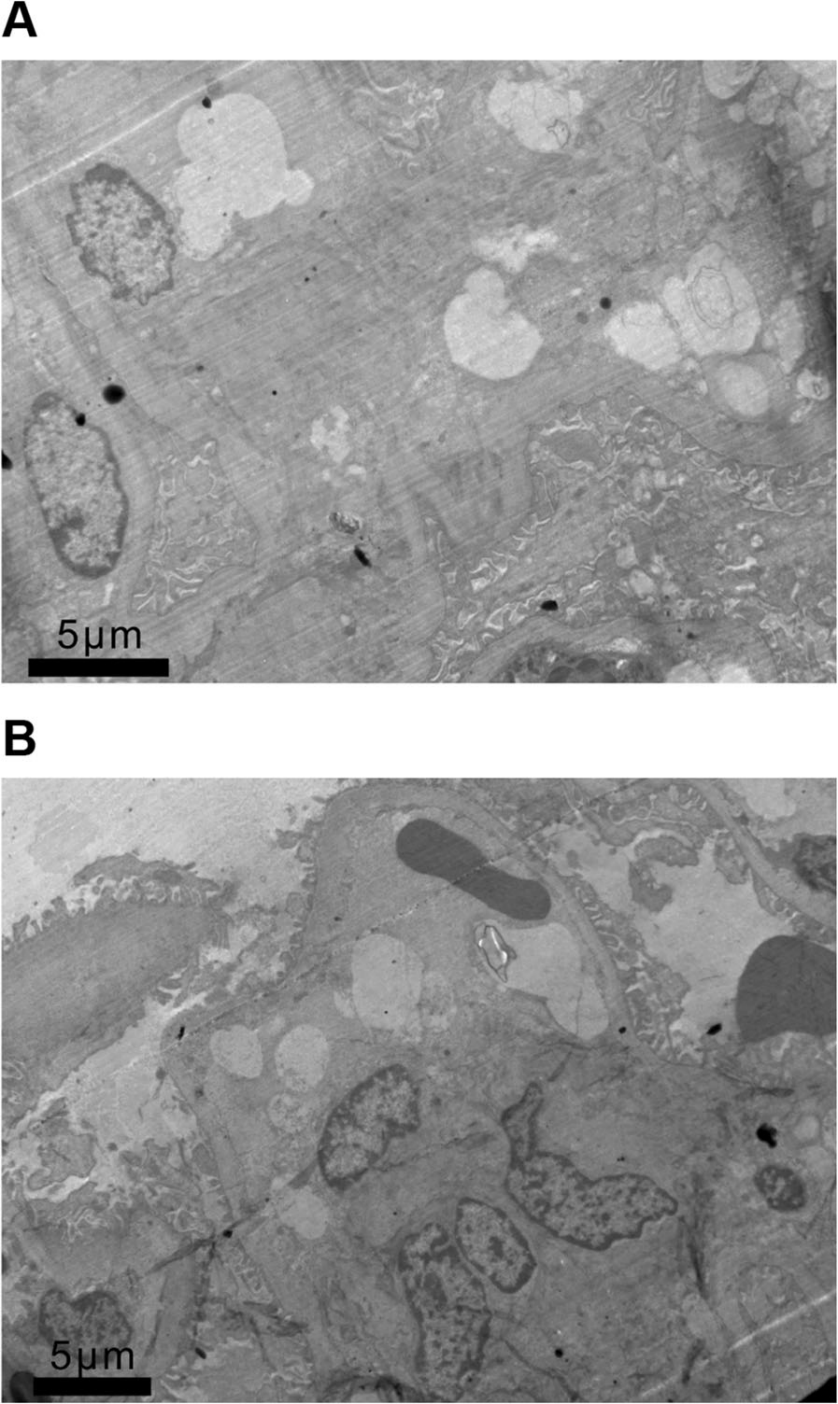
**a** Glomerular mesangial area diffuse severe enlargement with stromal hyperplasia, basal membrane diffused thickening, flexion, collapse and shrinkage (TEM × 1200). **b** Mesangial mesangial cells and matrix hyperplasia, capillary lumen incontinence, focal stenosis, collapse and occlusion, local foot process fusion (TEM × 1000)

Samples from one patient were further examined by electron microscopy. Three glomeruli were seen under electron microscopy; two were spherically sclerotic and one was segmentally sclerotic. No electron dense deposition was found in the mesangial cells and mesangial matrix hyperplasia. Capillary cavities were unobstructed, local focal stenosis, collapse and occlusion, basilar diffuse thickening, flexion, collapse and shrinkage, foot process fusion with microvilli degeneration, Bowman’s capsule adhesion, thickening of vesicle wall, renal tubular atrophy; basilar thickening, epithelial cells mild edema degeneration, and the number of transparent tube types were observed (Fig. 3). The moderate renal interstitial fibrosis, moderate lymphoid, mononuclear and foam cell infiltration were also observed.

## Discussion

In this study, we reported on renal injury with similar pathological changes observed in patients coming from the same job occupation (same workplace), without previous history of kidney disease and diabetes, who were exposed to CS2 for several years (working age about 13.2 years; 10 h per day; no protection, other than ordinary face mask) to CS2. All cases had moderate to severe nodular mesangial hyperplasia with renal tubular atrophy/interstitial fibrosis with less to moderate chronic inflammatory cell infiltration, as well as renal arteriosclerosis. In addition, no specificity for IgA, IgM, C3d, C4d, C1q, Fib were observed. After excluding other lesions, the final diagnosis suggested CS2-related idiopathic diffuse nodular mesangial hyperplasia - sclerosing nephropathy.

Following the unprecedented growth of Chinese economy, occupational hazards have received increasing attention. A number of studies [10–12] have shown that long-term exposure to CS2 can lead to hypertension, nervous system abnormality, ocular fundus damage, cerebral arteriosclerosis; cognitive dysfunction and mental decline [13]. In addition, a long-term exposure to CS2 has shown to induce renal function injury to some extent [14]. Although the clinical toxicity of CS2 has been studied for more than 100 years, the data reporting the association between CS2 and renal injury are very limited [15–18]. So far, only one study reported the clinical and pathological features in 10 patients with CS2 [19], which consequently resulted in renal injury.

In this study, there were nine cases of occupational renal damage caused by CS2, and there was no unified treatment plan. All the patients received standardized treatment according to the clinical practice guidelines of KDIGO glomerulonephritis; Six patients were treated with ACEI or ARB, Including the two patients with fiber crescent. While two patients who with cellular crescents received immunosuppressant medications; consequently, all patients required hemodialysis and renal transplantation. All patients were separated from their original working environment after onset, and were followed up for 10–50 months. Two patients developed end-stage renal disease, while the mental and nervous system symptoms did not decrease after symptomatic treatment in 2 cases. After dialysis, the mental symptoms improved in 1 case, the pain was relieved in both lower limbs, and the claudication was slightly reduced.

Through the description of clinical manifestations, pathological features, etiology and prognosis of 9 cases, the pathologic manifestations of CS2-related renal damage and the hazard of CS2 occupational exposure were recognized. The prognosis of CS2-induced renal damage was poor, the renal function of the patients continued to deteriorate, and the effect of clinical treatment was poor. Nevertheless, there were only 9 cases in this study, thus the description of the clinical and pathological features of CS2-related renal damage may be relatively limited. In addition, it remains unclear whether CS2 may cause liver damage. Thus, larger sample studies are required to further explore the effect of CS2 on humans.

## Conclusion

We speculate that nodular mesangial hyperplasia with type “K-W nodules” are characteristic pathological changes in renal disulfide kidney damage, but accurate conclusions require need further larger sample studies.

## Data Availability

The datasets used and/or analyzed during the current study are available from the corresponding author on reasonable request.

## Abbreviation

CRF: Chronic renal failure
CS2: Carbon disulfide
GN: Glomerulonephritis
IHC: Immunohistochemistry
